# Genome Sequences of Six Cluster N Mycobacteriophages, Kevin1, Nenae, Parmesanjohn, ShrimpFriedEgg, Smurph, and SpongeBob, Isolated on Mycobacterium smegmatis mc^2^155

**DOI:** 10.1128/MRA.00399-19

**Published:** 2019-05-30

**Authors:** Russell A. Caratenuto, Grace O. Ciabattoni, Nicolas J. DesGranges, Cassidy L. Drost, Longhui Gao, Brianna Gipson, Nicholas C. Kahler, Nicole A. Kirven, Julia C. Melehani, Krishna Patel, Alecia B. Rokes, Ryan A. Seth, Matthew C. West, Alexa A. Alhout, Francis F. Akoto, Nicole Capogna, Netta Cudkevich, Lee H. Graham, Matthew S. Grapel, Maaz M. Haleem, Jamie B. Korenberg, Brooke P. Lichak, Lauren N. McKinley, Kourtney R. Mendello, Caitlin E. Murphy, Lauren M. Pyfer, Wascar A. Ramirez, Julia R. Reisner, Rachel H. Swope, Matthew J. Thoonkuzhy, Lauren A. Vargas, Croldy A. Veliz, Katherine R. Volpe, Kevin D. Zhang, Dylan Z. Faltine-Gonzalez, Caitlin M. Zuilkoski, Catherine M. Mageeney, Hamidu T. Mohammed, Margaret A. Kenna, Vassie C. Ware

**Affiliations:** a Department of Biological Sciences, Lehigh University, Bethlehem, Pennsylvania, USA; Queens College

## Abstract

The annotation of six cluster N Mycobacterium smegmatis phages (Kevin1, Nenae, Parmesanjohn, ShrimpFriedEgg, Smurph, and SpongeBob) reveals regions of genomic diversity, particularly within the central region of the genome. The genome of Kevin1 includes two orphams (genes with no similarity to other phage genes), with one predicted to encode an AAA-ATPase.

## ANNOUNCEMENT

Unique mechanisms of immunity have been discovered for cluster N mycobacteriophages (1). Continued exploration of new cluster N phage genomes may reveal additional novel genes, some of which may be implicated in prophage-mediated defense systems.

Mycobacteriophages were isolated by enrichment from soil on host Mycobacterium smegmatis mc^2^155. Soil samples were placed in 7H9 medium with the host and incubated for 3 days at 37°C, and supernatants were sterile filtered for testing on host lawns. After purification and amplification of the phages, electron microscopy determined a *Siphoviridae* morphology for each of them. Sequencing, assembly, and finishing of the genomes were performed according to Russell (2). Briefly, sequencing libraries were prepared from genomic DNA (isolated by phenol-chloroform extraction) using an Ultra II FS kit (NEB) with dual-indexed barcoding and pooled with others for 48 total libraries run on an Illumina MiSeq instrument, yielding at least 482,600 single-end 150-base reads and at least 201-fold coverage for each genome. Reads were assembled using Newbler version 2.9 (3) with default settings, yielding a single phage contig for each. Consed version 29 (4) was used to check for completeness, accuracy, and genomic termini. The genomic characteristics are summarized in Table 1.

**TABLE 1:** Characteristics of cluster N genomes

Phage name	Soil isolation source	Genome size (bp)	Genome terminus (13-base 3′ overhang)	GC content (%)	No. of ORFs
Kevin1	Naugatuck, CT	41,988	5′-CCCGCCGCCCTCG	66.2	69
Nenae	Fairfield, NJ	42,597	5′-CCCGCCGCCTTGG	66.1	70
Parmesanjohn	Bethlehem, PA	43,700^a^	5′-CCCGCCGCAATGG	66.3	72
ShrimpFriedEgg	Bethlehem, PA	42,594	5′-CCCGCCGCCTTGG	66.1	70
Smurph	Manchester, NH	43,700^a^	5′-CCCGCCGCAATGG	66.3	72
SpongeBob	La Cañada Flintridge, CA	41,287	5′-CCCGCCGCCTTGG	66.2	65

a Although the Parmesanjohn (isolated in 2017) and Smurph (isolated in 2018) genomes differ by only 2 nucleotides, the geographic location and year of isolation make cross-contamination unlikely.

DNA Master (5), embedded with GeneMark 2.5 (6) and GLIMMER 3.0 (7), was used to annotate the open reading frames (ORFs). NCBI BLASTP 2.7, NCBI’s conserved domain database (8), and HHpred (9) were used to assign protein functions. Phamerator (10) was used for comparative genomic analysis.

The genomic architecture conforms to that of other cluster N genomes (1). Conserved structural assembly genes are located in the left arm, followed by a central variable region and a diverse right arm that includes DNA maintenance genes (1). The lysis cassette in the left arm encodes lysin A and lysin B in Kevin1; other genomes (reported here) encode only lysin A. One tRNA was predicted in SpongeBob and Parmesanjohn using ARAGORN (11) and tRNAscan (12) but was not annotated, because its location disrupts the leftward transcribed immunity repressor ORF. The central variable regions of Parmesanjohn, Smurph, and SpongeBob share 97% average nucleotide identity (ANI), while Nenae, ShrimpFriedEgg, and Redi (GenBank accession number JN624851) share 100% ANI. The unique variable region of Kevin1 contains two orphams (*30* and *31*), of which gene *30* is predicted to encode an AAA-ATPase. Functional characterization of these orphams may offer additional insights into cluster N immunity mechanisms.

### Data availability.

The GenBank and NCBI SRA database (for raw reads) accession numbers, respectively, are MK524500 and SRX5572872 (Kevin1), MK524520 and SRX5572867 (Nenae), MK524515 and SRX5572865 (Parmesanjohn), MK524528 and SRX5572863 (ShrimpFriedEgg), MK524518 and SRX5572864 (Smurph), and MK524509 and SRX5572876 (SpongeBob).
